# Patient and public involvement and engagement: Mind the gap

**DOI:** 10.1111/hex.12962

**Published:** 2019-09-11

**Authors:** Gary Hickey, Mary Chambers

**Affiliations:** ^1^ University of Southampton Science Park Southampton UK; ^2^ Kingston University London UK; ^3^ St. George's University of London London UK

Let us begin with a cautionary tale—Mind the Gap! There is a serious message behind the warning “Mind the Gap” that we hear when travelling on the London Underground—a reminder to passengers that when alighting from a train, there is a gap between train and platform. This warning also applies to patient and public involvement and engagement across the health and social care trajectory as illustrated in this edition of HEX. The papers highlight the gap between patient and public involvement and engagement (PPIE) policy and practice in service design, research and education. Having a special issue of HEX devoted to patient and public involvement and engagement (PPIE) illustrates the importance of acknowledging and promoting PPIE across all areas of health and social care both nationally and internationally.

Several authors, for example, Boylan et al, Puerta et al, and Troya et al, note how patient and public involvement and engagement (PPIE) is increasingly required by research funders. There is also a growing recognition of the importance and benefit of PPIE when designing care services as reported in the paper by Hertel et al Indeed, patients and public have the right to be involved in research, education and the design of services that impact upon them.^1^

Greenhalgh et al identify a burgeoning set of frameworks to aid PPIE efforts but questioned their transferability and suggest that a single “off‐the‐shelf” framework may be of limited value to stakeholders and more would be gained from “*using evidence‐based resources to co‐design their own frameworks*”. As previously indicated by other authors, effective PPIE can lead to better and more ethically sound research.^2^

A key theme in many papers in this edition is “co‐production”—an approach to research and service design that is gaining traction and puts the public and patients at the heart of decision making. It involves public, researchers and professionals working together and sharing power in decision making.^3^ Co‐production, with its emphasis on the development of relationships, being inclusive, respecting and valuing the knowledge of all those working together, has the potential to contribute towards the rebuilding of trust between the public on the one hand and researchers and professionals on the other.

Many of the papers show how difficult it is to share power across the spectrum of health and social care and highlight the educational and professional challenges faced by the professional contingent. There are often power differentials between the public and researchers. This is particularly so when the focus is on groups, perhaps considered as marginalized or seldom heard. These groups include people with autism in the UK Somali community (Aabe et al); the LGBTI community (Sherriff et al); people with traumatic brain injury (Makela et al); those experiencing dementia (Waite et al); children and young people (Alderson et al, and Pavarini et al); people with mental health issues (Gault et al, and King and Gillard); people with learning disabilities (Cook et al); and older adults (Troya et al).

Many of the papers in this special issue have identified several challenges when meaningfully engaging with patients and public. These challenges include the cultures of organizations and their processes, professional identity and the desire to retain professional supremacy and power. Worth noting is the challenge to funders to ensure that projects are given adequate time and funding to enable PPIE (Waite et al).

Professional and research cultures, academic structures and career development can all mitigate against the sharing of power as indicated by Boylan et al and Troya et al The nature and forms of knowledge present further challenges as the value placed on experiential knowledge still does not receive parity with that of professional or research knowledge as suggested by Cook et al and Puerta et al

Other factors of note for effective PPIE are that public and patients need to be adequately supported by the professionals and researchers with whom they are working (Barber et al, Giebel et al Sutton et al). McCarron et al acknowledge that professional staff require support by their organizations and point to the need to understand the motivations of people who wish to get involved and to learn from the experiences of those who have been involved. A number of papers (Aabe et al, Goold et al, Noyes et al, and Stallings et al) all note the perennial challenge of demonstrating the impact of the efforts to include patients and public.

The papers in this issue of HEX are both a celebration of PPIE initiatives and the growing influence of co‐production. However, they are also a timely reminder of the challenges posed by organizational cultures and processes as well as professional approaches to ensure that the gap between rhetoric and reality is bridged.
